# Cytoplasmic-targeted parvalbumin blocks the proliferation of multipotent mesenchymal stromal cells in prophase

**DOI:** 10.1186/scrt291

**Published:** 2013-08-08

**Authors:** Carolina Soares Barros Melo, Jerusa Araújo Quintão Arantes Faria, Natássia Caroline Resende Corrêa, Carolina de Andrade, Juliana Lott Carvalho, Alfredo M Goes, Michele A Rodrigues, Dawidson Assis Gomes

**Affiliations:** 1 Department of Biochemistry and Immunology, Universidade Federal de Minas Gerais, Av. Antonio Carlos 6627, Belo Horizonte-MG ZIP Code: 31270-901, Brazil

**Keywords:** Mesenchymal stem cells, Targeted parvalbumin, Calcium signaling, Proliferation, Cyclins

## Abstract

**Introduction:**

Multipotent mesenchymal stromal cells (MSCs) have gained considerable interest because of their potential use in the treatment of a variety of diseases and injuries. Although remarkable advancements have been made in clinical studies, substantial concerns still regard the safety of MSCs. Some evidence suggests that MSCs can spontaneously generate a population of cells with tumorigenic potential. Thus, studying the molecular mechanisms that control the proliferation of MSCs may be a necessary step toward the development of strategies for safe clinical practice. Ca^2+^ is a second messenger that mediates a wide range of cellular responses, including the regulation of cell proliferation, but little is known about its function in MSCs. The aim of this study was to investigate the effects of targeted Ca^2+^ buffering on MSCs proliferation *in vitro*.

**Methods:**

Here, we used an adenoviral (Ad) vector encoding the Ca^2+^ chelator protein parvalbumin (PV) fused to a nuclear exclusion signal (NES) and the *Discosoma* red fluorescent protein (DsRed) to investigate the function of cytoplasmic Ca^2+^ signals on MSC proliferation. Confocal microscopy was used to demonstrate that PV-NES-DsRed was expressed in the cytoplasm. Ca^2+^ signaling was monitored by using Fluo-4-AM. Fluorescence-activated cell sorting (FACS) analysis of cells that were stained with propidium iodide was used as a quantitative measure of cell death. The mitotic index was assessed by immunofluorescence, and the expression of cyclins was examined with Western blot.

**Results:**

Our results show that the Ad-PV-NES-DsRed fusion protein decreased serum-induced Ca^2+^ signaling and blocked the proliferation of rat adipose-derived MSCs (AT-MSCs) in prophase. FACS analysis revealed that Ad-PV-NES-DsRed did not induce cell death in AT-MSCs. Furthermore, Western blot analysis demonstrated that Ad-PV-NES-DsRed reduced extracellular signal-regulated kinase (Erk1/2) phosphorylation and cyclin B1 expression. Buffering cytosolic Ca^2+^ did not alter the expression of cyclins A/D1/D2/D3/E and E2.

**Conclusions:**

Our results show that cytoplasmic Ca^2+^ signals are important for AT-MSCs progression beyond prophase because of their effects on Erk phosphorylation and cyclin B1 expression.

## Introduction

Multipotent mesenchymal stromal cells (MSCs) are nonhematopoietic stromal cells that have generated a great amount of interest in the field of regenerative medicine because of their unique biological properties. These cells give rise to diverse tissues, including bone, cartilage, tendon, muscle, and adipose tissue [1], and they have been isolated from different sources, such as bone marrow, adipose tissue, peripheral blood, muscle, umbilical blood, placenta, and other sites [2,3]. These cells are relatively easy to obtain and have a remarkable capacity for extensive *in vitro* expansion, which allows them rapidly to reach the cell number required for *in vivo* therapy. In addition to their secretion of multiple bioactive molecules with trophic effects, MCSs are able to migrate and to exert immunomodulatory activities [4].

Over the past decade, many publications on MSCs reported experimental and clinical applications for these cells and demonstrated encouraging results. Although tremendous advancements have been made in clinical studies, substantial challenges remain and must be overcome before MSCs therapy can fulfill its promise in wider clinical practice [5].

The first major obstacle is definitively to determine the safety of MSCs. A few studies support the idea that MSCs suppress tumor growth, whereas others state that MSCs may contribute indirectly to cancer by antiapoptotic effects that protect tumors and by the promotion of tumor progression, metastasis, and drug resistance. Alternatively, MSCs may be directly involved in cancer development through malignant transformation [6].

Some evidence suggests that these cells can spontaneously transform to generate a population of cells with tumorigenic potential through the acquisition of point mutations [7]. Some of these mutations are involved in the expression of molecules that regulate the cell cycle and cell proliferation [8]. Thus, studying the molecular mechanisms involved in the cell cycle and proliferation of MSCs may be the first step toward the development of control strategies that prevent the proliferation of MSCs that have undergone tumorigenic transformation after transplantation for safe clinical practice.

Ca^2+^ is a second messenger that contributes to the cell cycle and cell proliferation. In mammalian somatic cells, the importance of intracellular Ca^2+^ signaling during cell-cycle progression is well established [9], but little is known about the effects of Ca^2+^ signals in the cell cycle and proliferation of MSCs. The first studies in this area used the microinjection of dextran-linked Ca^2+^ buffers, but a more-efficient approach was subsequently developed that used the targeted expression of Ca^2+^-buffering proteins, such as parvalbumin (PV) or calretinin [10,11]. Here, we used a construct encoding PV that was targeted to the cytoplasm by a nuclear export signal (PV-NES), and we delivered these constructs to MSCs by using an adenovirus (Ad) expression system to achieve high-efficiency gene delivery. We used this strategy to examine the effects of targeted Ca^2+^ buffering on cell populations. The PV protein was fused to the *Discosoma* red fluorescent protein (DsRed) to monitor its expression and subcellular localization. The aim of this study was to investigate the effects of Ad-PV-NES-DsRed on MSCs proliferation *in vitro*.

We found that Ad-PV-NES-DsRed could block MSC proliferation during prophase because of its effects on Erk phosphorylation and cyclin B1 expression. Thus, this tool might provide a new perspective for understanding and controlling MSC proliferation.

## Methods

### Cell isolation and culture

Male Wistar rats (6 to 8 weeks old), which were obtained from the Centro de Bioterismo da Universidade Federal de Minas Gerais (CEBIO), were used for all of the studies. Rat inguinal adipose tissue was dissected into small pieces, digested with 0.15% collagenase B (Roche Applied Science, Indianapolis, IN, USA) in 0.1 *M* phosphate-buffered saline (PBS) for 40 minutes at 37°C. Mature adipocytes and connective tissues were separated from the cell pellet by centrifugation at 274.4 *g* for 10 minutes at room temperature. The cell pellet was resuspended in high-glucose Dulbecco modified Eagle medium (DMEM) (Sigma-Aldrich, St. Louis, MO, USA) with 10% fetal bovine serum (FBS) and 100 U/ml penicillin/streptomycin (Life Technologies, Carlsbad, CA, USA). The current protocol was adapted from a previously described protocol [12].

The initial passage of the primary cell culture was referred to as passage 0. We used cells in passage 3 only, except in the indicated experiments. The cells were maintained in complete media (DMEM + 10% FBS + penicillin/streptomycin) until they achieved 75% to 90% confluence. The cells were then replated in T75 tissue-culture flasks. The identity of the MSCs was defined by the criteria proposed by the International Society for Cellular Therapy [13]. The experiments were approved by the local ethical committee on animal experimentation (CETEA/UFMG; protocol number 239/10).

### Phenotypic analysis with flow cytometry

MSCs were recognized by using specific antibodies against CD54, CD73, and CD90. In addition, we assessed the lack of expression of the CD45 hematopoietic marker. All antibodies were purchased from Becton Dickinson (BD) Biosciences (San Jose, CA, USA). The secondary antibodies used were conjugated to Alexa Fluor 488 (Life Technologies).

For immunophenotypic analysis, the MSCs were detached by incubation with 0.05% trypsin-EDTA (Life Technologies) for 5 minutes, immediately resuspended in DMEM plus 10% FBS, and plated in 96-well culture plates at a density of 2 × 10^5^ cells/well. Next, the cells were washed with PBS, and the cell suspension (100 μl) was incubated at 4°C for 30 minutes with the primary antibody (1:50) or with an isotype antibody (1:50) as a negative control. Then, the plate was centrifuged at 274.4 *g* for 10 minutes at 10°C. After two washes with PBS, the cell suspension was incubated at 4°C for 30 minutes with the secondary antibody (1:200). Subsequently, the cells were washed with PBS and analyzed with FACScan flow cytometry by using CELLQuest software (BD Biosciences). All fluorescence-activated cell-sorting (FACS) data were analyzed by using FlowJo software (Tree Star, San Carlos, CA, USA).

### Adenoviral constructs

Adenoviral constructs encoding DsRed (Ad-DsRed) and the PV-DsRed fusion protein that was targeted to the cytosol (Ad-PV-NES-DsRed) were obtained as previously described [10]. The adenoviruses were amplified in HEK-293 cells, a cell line that constitutively expresses the E1 protein. Both viruses (Ad-DsRed and Ad-PV-NES-DsRed) were generated by using standard procedures and stored at −80°C in PBS plus 7% glycerol. Stocks of the adenoviral particles were quantified as plaque-forming units (pfus) by using plaque assays in HEK-293 cells. On day 1 of culture, each adenovirus construct (at a multiplicity of infection (MOI) of 100) was diluted in regular culture medium (DMEM) containing 10% FBS. The experiments were performed at 36 to 48 hours, when approximately 70% of the cells were transduced.

### Ca^2+^ measurements

MSCs were cultured in 96-well culture plates at a density of 1 × 10^4^ cells/well and then transduced with the adenoviral constructs. After 24 hours, the cells were synchronized to G_0_ by serum starvation overnight. The next morning, the cells were washed in PBS and resuspended in HEPES buffer containing 3 μ*M* Fluo-4-AM (Life Technologies) for 30 minutes at 37°C. Next, the cells were washed, resuspended in HEPES, and then stimulated with HEPES containing 10% FBS. The resultant fluorescent signal was monitored for the whole well over time by using a Synergy 2 multimode plate reader (BioTek, Winooski, VT, USA). The wavelength for excitation was 485/20 nm; emission was measured at 530/25 nm, and the dichroic that was used was the top 50%. The experiments were performed at 37°C. The mean peaks of the maximum and minimum fluorescence were analyzed by using Excel and plotted by using GraphPad software (La Jolla, CA, USA).

### Analysis of cell viability

To assess the cell viability of the MSCs, we used the 3-(4,5-dimethylthiazol-2-yl)-2,5-diphenyltetrazolium bromide (MTT) assay (Life Technologies), as previously described [14]. In brief, the MSCs were plated in 24-well culture plates at a density of 1 × 10^5^ cells/cm^2^ per well. MTT (5 mg/ml) was added to each well of the monolayer cultures, and the cultures were incubated in a humidified 5% CO_2_ incubator at 37°C. Two hours later, the cell morphologies and formazan salts were visualized by using an inverted optical microscope. The formazan salts were dissolved with 10% SDS-HCl overnight, and the optical density was measured at 595 nm by using a microplate reader (Elx800; BioTek). Staurosporine was used as a positive control for cell death at a concentration of 100 n*M* in DMEM containing 10% FBS. The values from the samples are expressed as percentages of the control. All data were analyzed by using GraphPad software.

### Analysis of cell death by flow cytometry

A flow-cytometric DNA fragmentation assay was used as a quantitative measure of cell death [15]. Forty-eight hours after transducing the MSCs with the adenoviral constructs, the cells were collected by centrifugation and lysed with 300 μl of a hypotonic solution containing 0.1% sodium citrate, 0.1% Triton X-100, and 20 μg/ml propidium iodide (PI; Life Technologies). Then the cells were incubated at room temperature for 1 hour and analyzed with a FACScan flow cytometer (BD Biosciences) for shifts in PI fluorescence, which are indicative of nuclei with hypodiploid DNA content. All data were analyzed by using FlowJo software (Tree Star).

### Mitotic index measurements by immunofluorescence

Confocal immunofluorescence was performed as previously described [10,16]. In brief, the cells were labeled with an anti-phospho-histone-3 monoclonal antibody (1:500; Millipore, Billerica, MA, USA) and then incubated with an Alexa Fluor 488-conjugated secondary antibody (1:1,000; Life Technologies) and the Hoechst nuclear stain (200 ng/ml; Life Technologies). Images were captured by using a Zeiss LSM 510 confocal microscope with a 63×/1.4 NA objective lens. The samples were excited at 488 nm and observed at 505 to 550 nm to detect Alexa Fluor 488, excited at 405 nm, and observed at 420 to 460 nm to detect Hoechst staining. Mitotic indexes were scored for the cells in each phase of mitosis according to the phospho-histone-3 distribution and DNA condensation pattern [10].

### Immunoblotting

Immunoblotting was performed by using standard methods [10,16]. In brief, the cells, which were grown in T75 tissue-culture flasks, were washed 3 times with PBS and lysed in buffer containing 150 m*M* NaCl, 1 m*M* EDTA, 20 m*M* Tris–HCl, 0.5% Nonidet P-40, and a protease-inhibitor mixture (Sigma-Aldrich). The protein concentration was determined spectrophotometrically by using the Bradford method (Sigma-Aldrich), and 40 μg of protein was separated by electrophoresis in a 4% to 15% polyacrylamide gel and then transferred to a polyvinylidene difluoride membrane (Millipore). The membrane was blocked with 5% skim milk in Tris-buffered saline (TBS) plus 0.5% Tween 20 (TBST) for 60 minutes and then incubated with primary antibodies against total Erk1/2 or phospho-Erk1/2 (1:1,000; Cell Signaling Technology, Beverly, MA, USA), cyclins A, B1, D1, D2, D3, E, or E2 (1:500 to 1,000, Cell Signaling Technology) or GAPDH (1:500; Santa Cruz Biotechnology, Santa Cruz, CA, USA). The incubations with the primary antibodies were carried out overnight. After three 5-minute washes with TBST, the membranes were incubated with the appropriate horseradish peroxidase-conjugated secondary antibody (1:5,000) (Sigma-Aldrich) for 1 hour at room temperature. After three additional 5-minute washes with TBST, the membranes were developed by using enhanced chemiluminescence (ECL Plus; GE Healthcare Life Sciences, Piscataway, NJ, USA). Subsequently, the films were scanned and analyzed by using Image J software.

### Statistical analysis

All of the data shown represent at least three independent experiments and are expressed as the mean ± standard error of the mean (SEM). The statistical analyses were performed with GraphPad software. The experiments with more than two data groups were compared by using one-way ANOVA and the Bonferroni posttest. *P* values of <0.05 were considered to be statistically significant.

## Results

### Characterization of rat MSCs

**Figure 1:**
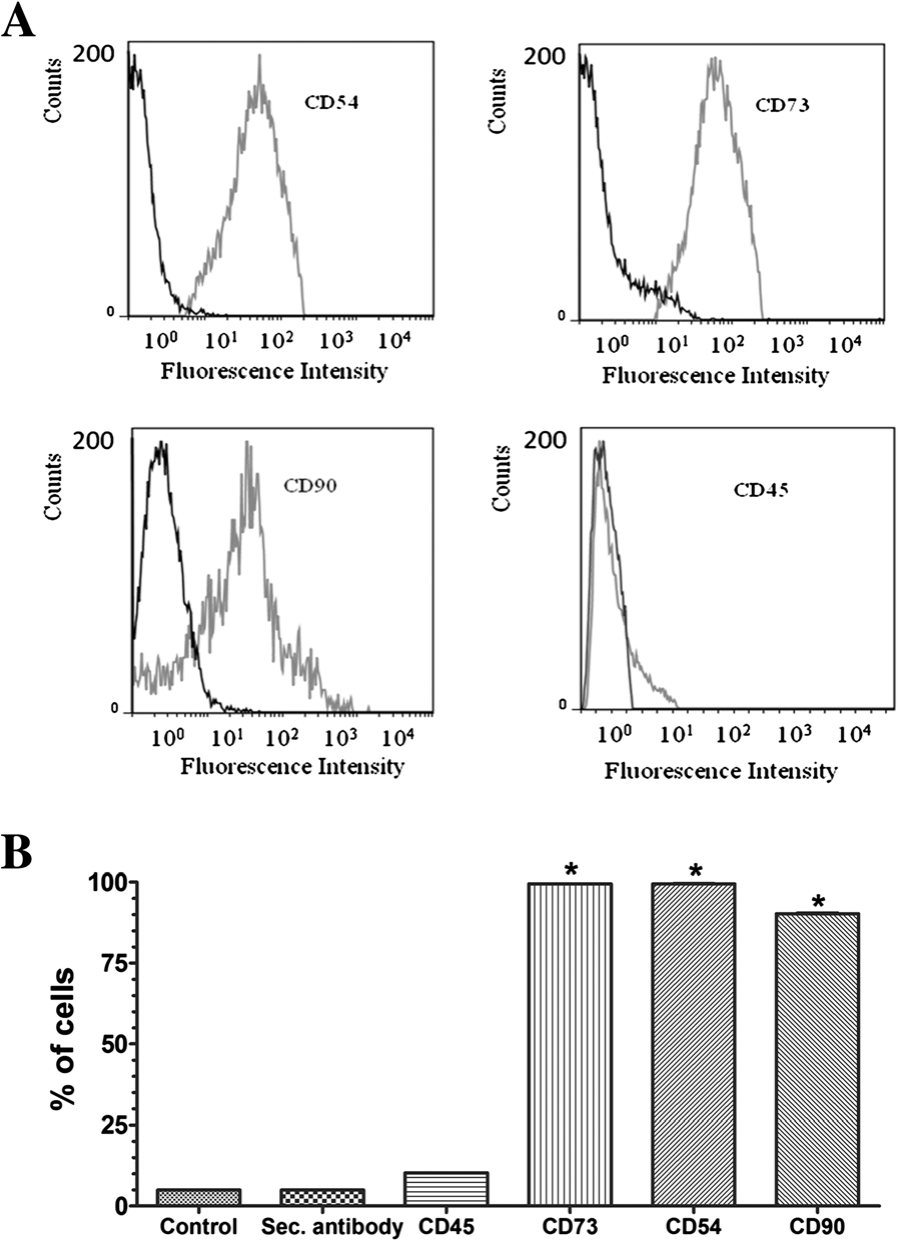
**Phenotypic analysis of rat MSCs. (A)** FACS analysis of CD45, CD54, CD73, and CD90 antigens in rat MSCs (gray bars). Solid black bars refer to the negative controls (cells incubated with isotype IgG control and secondary antibody). **(B)** Quantitative analysis of the expression pattern of rat MSCs. The control group comprised cells that were not incubated with the Alexa Fluor 488-conjugated secondary antibody. The secondary antibody (sec. antibody) group comprised cells that were incubated with the isotype IgG control and secondary antibody alone. *P < 0.001 compared with control; *N* = 3 (three experiments that were conducted independently).

Rat MSCs were characterized according to the three criteria proposed by the Mesenchymal and Tissue Stem Cell Committee of the International Society for Cellular Therapy [13]. First, the cell-surface antigen profile was ascertained by staining the cells with rat-specific monoclonal antibodies, followed by flow-cytometric analyses, as shown in Figure 1. We investigated the expression of the cell markers CD54 (99.1% ± 0.4%), CD73 (99.5% ± 0.2%), CD90 (90.3% ± 0.6%), and CD45 (10.3% ± 0.3%). Second, we demonstrated that the MSCs were plastic-adherent when maintained in standard culture conditions, and fibroblast-like, as they appeared polygon-like or spindle-like with processes (data not shown). Third, we showed that the MSCs were capable of differentiating into osteoblasts and adipocytes (data not shown).

### Cytoplasmic-targeted parvalbumin decreases agonist-induced Ca^2+^ signals

**Figure 2:**
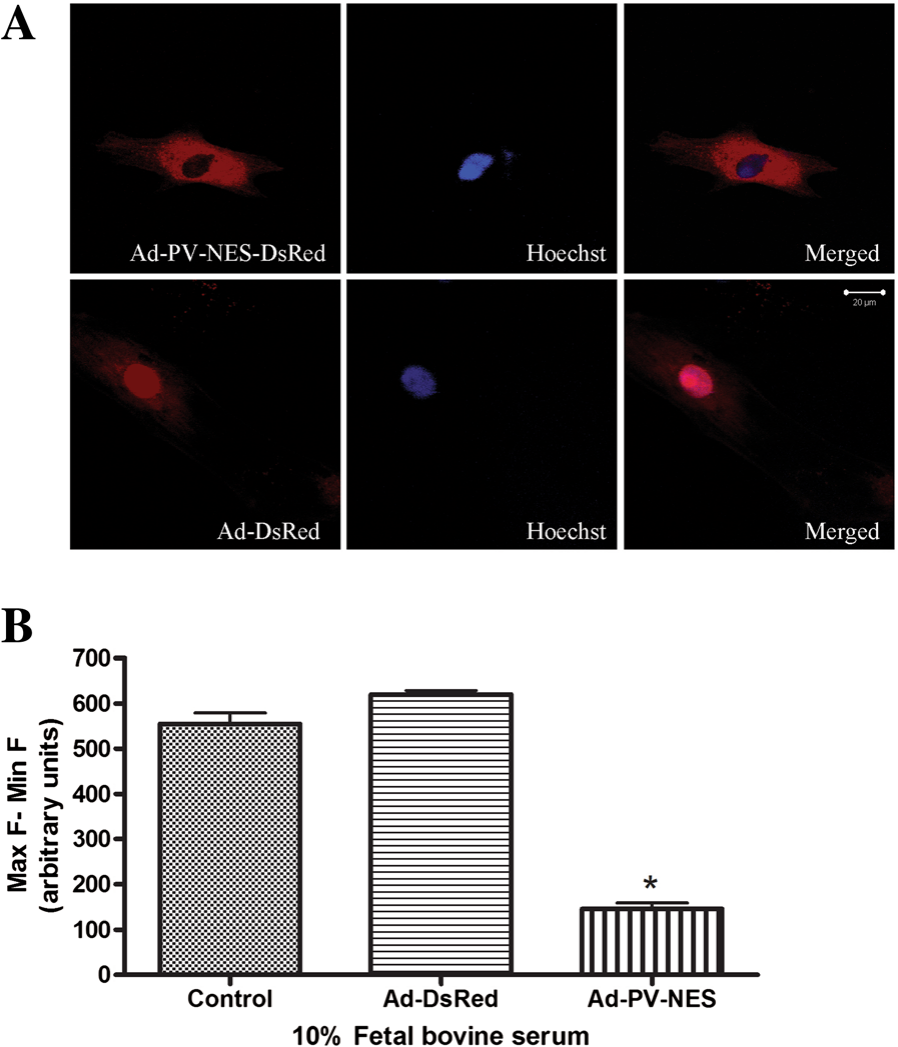
**Parvalbumin attenuates serum-induced Ca**^**2+ **^**signaling in MSCs. (A)** Analysis of parvalbumin protein localization by confocal microscopy. Ad-PV-NES-DsRed was expressed in the cytoplasm, and ad-DsRed alone, which served as a control, was expressed in both compartments. In each image, red indicates DsRed, blue indicates Hoechst nuclear staining, and purple represents the colocalization of the two signals. **(B)** Effect of parvalbumin on Ca^2+^ signaling. The cells were examined 48 hours after being transduced with the indicated adenoviral constructs and were synchronized to G_0_ by serum starvation overnight. Ca^2+^ was monitored with Fluo-4-AM by using a fluorescence microplate reader in cells that were stimulated with 10% serum. Ad-PV-NES-DsRed (Ad-PV-NES) attenuated the serum-induced increase in Ca^2+^ (*P* < 0.05). Ca^2+^ signals were not attenuated in cells that expressed ad-DsRed alone (*P* > 0.05). The results are representative of three independent experiments. Scale bar, 20 μm.

The relative role of cytoplasmic Ca^2+^ signals in specific cell functions can be determined by selectively attenuating Ca^2+^ increases in the cytoplasm with Ad-PV-NES-DsRed, as shown previously [10,17]. In this study, confocal microscopy demonstrated that PV-NES-DsRed was expressed in the cytoplasm and that DsRed alone was expressed in both the cytoplasm and nucleus (Figure 2A). We used 10% serum stimulation to test the efficiency of Ad-PV-NES-DsRed in attenuating agonist-induced Ca^2+^ signaling in MSCs. The expression of Ad-PV-NES-DsRed was able to attenuate locally 74% ± 2% of the cytoplasmic Ca^2+^ signals (Figure 2B).

### Cytosolic Ca^2+^ blocks MSC proliferation at prophase

**Figure 3:**
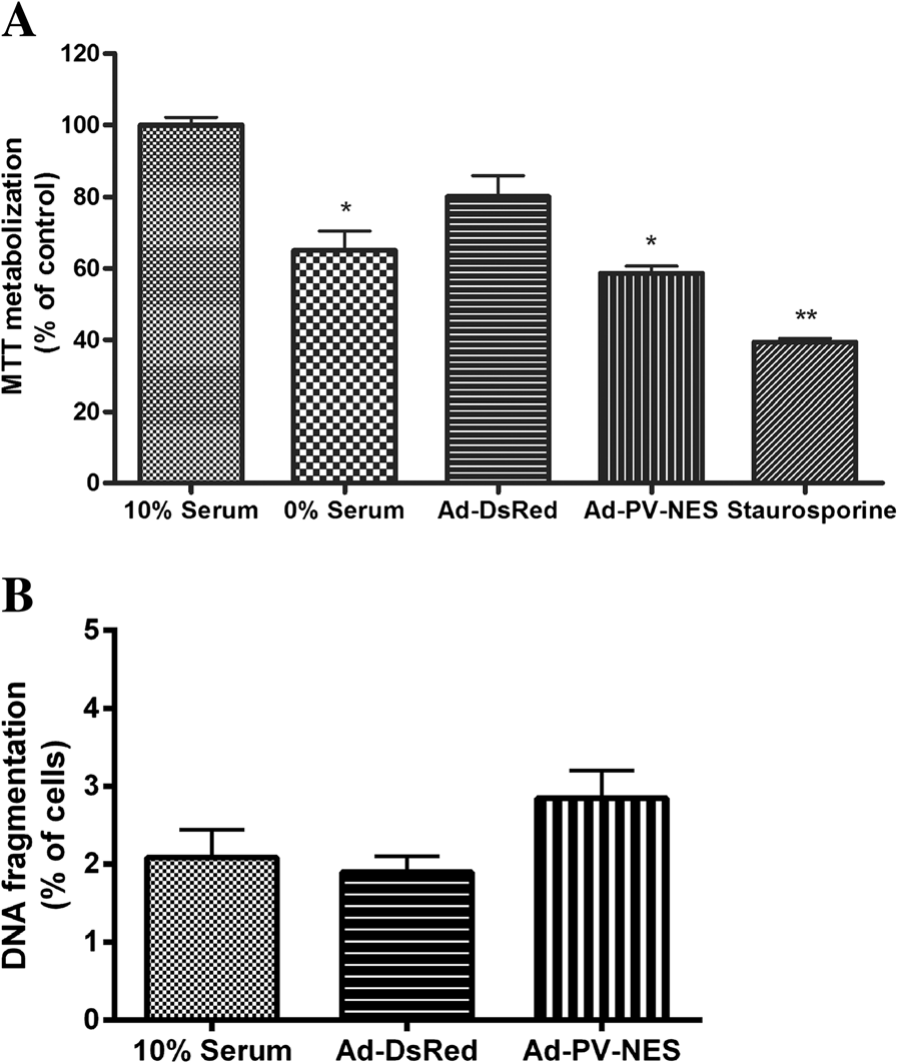
**Parvalbumin targeted to the cytoplasm specifically inhibits MSC proliferation. (A)** MTT assay of MSCs after 48 hours of transduction with Ad-PV-NES-DsRed. The metabolization of MTT was reduced in cells that were transduced with Ad-PV-NES-DsRed compared with cells grown with serum (*P* < 0.01), whereas no reduction in the metabolization of MTT was observed in cells that expressed the adenoviral transduction control Ad-DsRed compared with cells grown with serum (*P* > 0.05) (*n* = 3). **(B)** Flow-cytometric assays were used to measure cell death quantitatively after cells were stained with PI. The cells were examined 48 hours after transduction. The results are expressed as the percentage of events from a total of 20,000 events (*n* = 3). *P < 0.01 compared with cells grown in 10% serum. **P < 0.05 cells grown without serum compared with cells treated with 200 n*M* staurosporine.

To determine the involvement of cytoplasmic Ca^2+^ in MSC proliferation, the effect of the expression of our Ca^2+^ chelator fusion protein, PV-NES-DsRed, in MSCs was examined. First, the cells were synchronized to G_0_ by serum starvation and then transduced with the adenoviral constructs. Next, the cells were stimulated with complete medium to reenter the cell cycle. After 48 hours, the metabolization of MTT was reduced in cells that were transduced with Ad-PV-NES-DsRed compared with cells grown without serum, whereas no reduction in the metabolization of MTT was observed in cells that expressed the adenoviral transduction control Ad-DsRed compared with cells grown with serum (Figure 3A). Furthermore, flow-cytometric analysis of cells stained with propidium iodide showed that Ad-PV-NES had no effect on cell death (Figure 3B). These results suggest that the effects of Ad-PV-NES were specific to MSCs proliferation.

**Figure 4:**
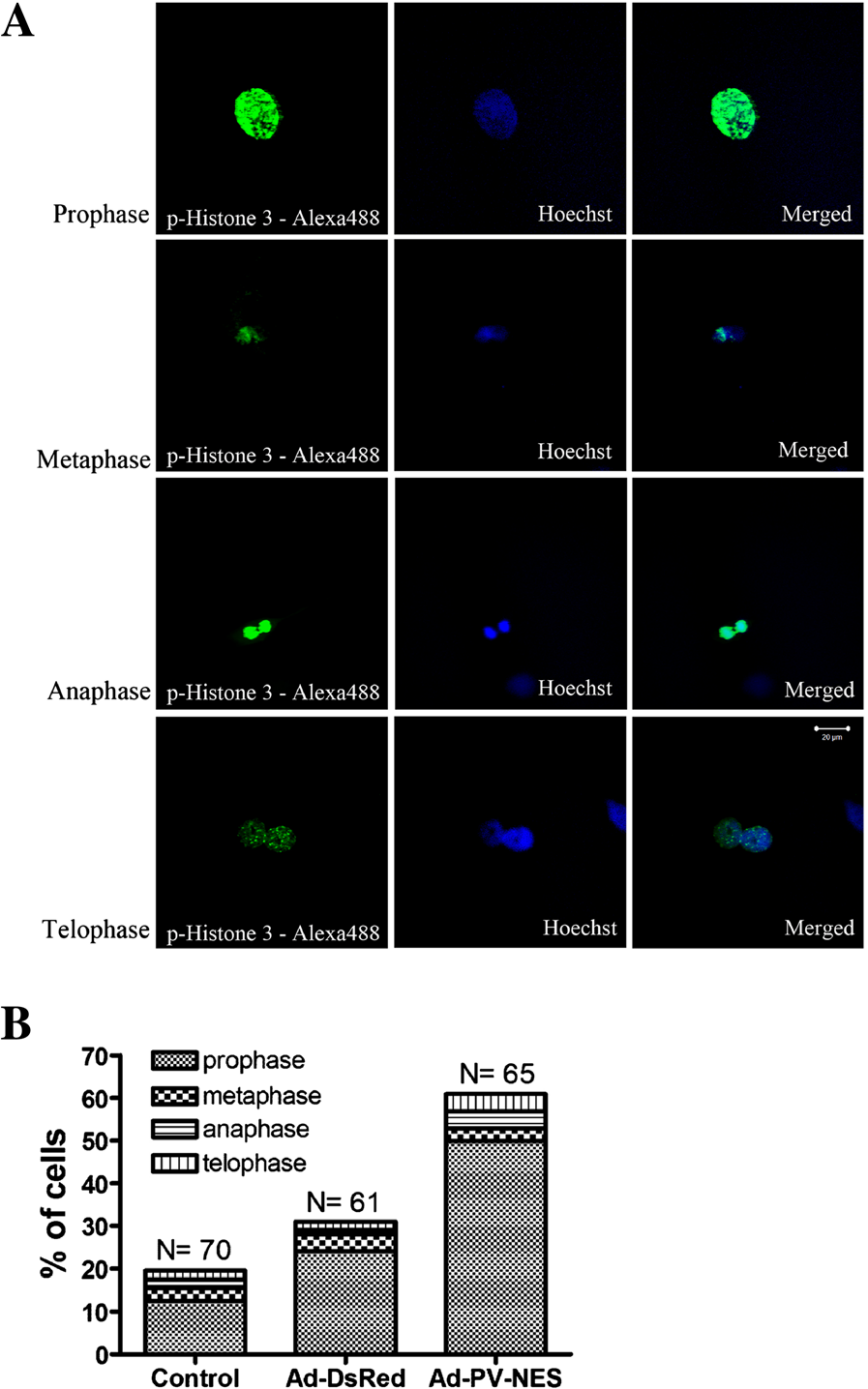
**Cytosolic Ca**^**2+ **^**regulates the progression of MSCs through prophase. (A)** Representative images of MSCs in prophase, metaphase, anaphase, and telophase. The confocal immunofluorescent images were obtained after staining with anti-phospho-histone-3 (green) and Hoechst dye (blue) to label the nucleus. Scale bar = 20 μm. **(B)** Mitotic MSCs were identified by phospho-histone-3 labeling and measured 48 hours after transduction with the indicated adenoviral constructs. Approximately 10 random fields were analyzed by using an Olympus IX70 inverted epi-fluorescent microscope to determine the average number of cells in the samples (61 to 70 cells were analyzed in each experimental group). The mitotic index increased to 61% ± 2% in cells in which cytosolic Ca^2+^ was buffered (*P < 0.001). In addition, 49.9% of the mitotic cells were in prophase.

**Figure 5:**
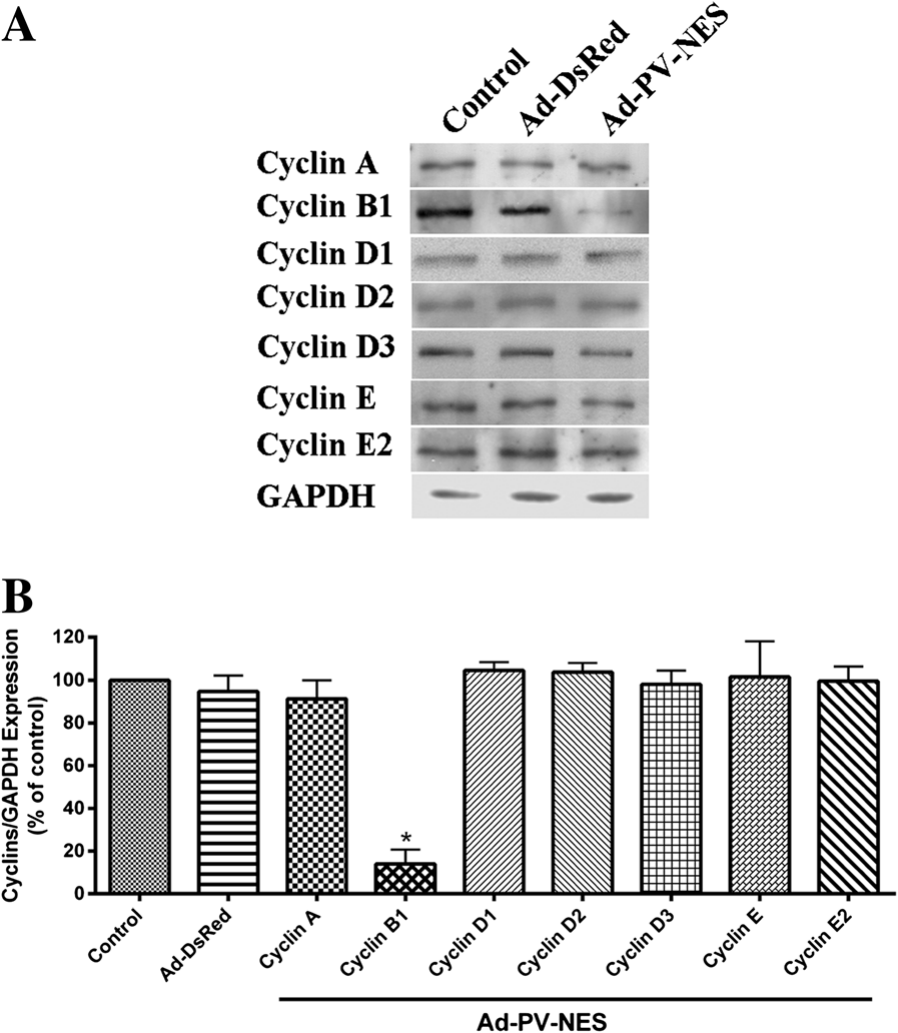
**Cytosolic Ca**^**2+ **^**regulates expression of cyclin B1. (A)** Immunoblot analysis of cells that were transduced with the Ad-PV-NES-DsRed construct. The expression of cyclin B1 was decreased in the presence of the PV-NES-DsRed protein, but no changes were observed in the expression of the cell-cycle checkpoint proteins cyclins A, D1, D2, D3, E, or E2. GAPDH was used as a loading control. **(B)** Bar-graph summary showing that Ad-PV-NES-DsRed decreased the expression of cyclin B1 by 86% ± 7% (*n* = 3). *P < 0.05.

Second, to investigate the effects of cytoplasmic Ca^2+^ on mitosis, the MSCs were labeled with the mitotic marker phospho-histone-3. After 48 hours of adenoviral transduction, the cells were examined with confocal microscopy to determine the fraction of cells in mitosis (Figure 4B). The mitotic index was increased to 61% ± 2% in cells with buffered cytoplasmic Ca^2+^ (*P* < 0.001). In addition, 49.9% of the cells in mitosis were in prophase (Figure 5B). No significant effect was observed upon comparison of uninfected cells with Ad-DsRed-transduced cells (Figure 4B). These results demonstrate that cytoplasmic Ca^2+^ is important for the cell-cycle transition of MSCs beyond prophase.

**Figure 6:**
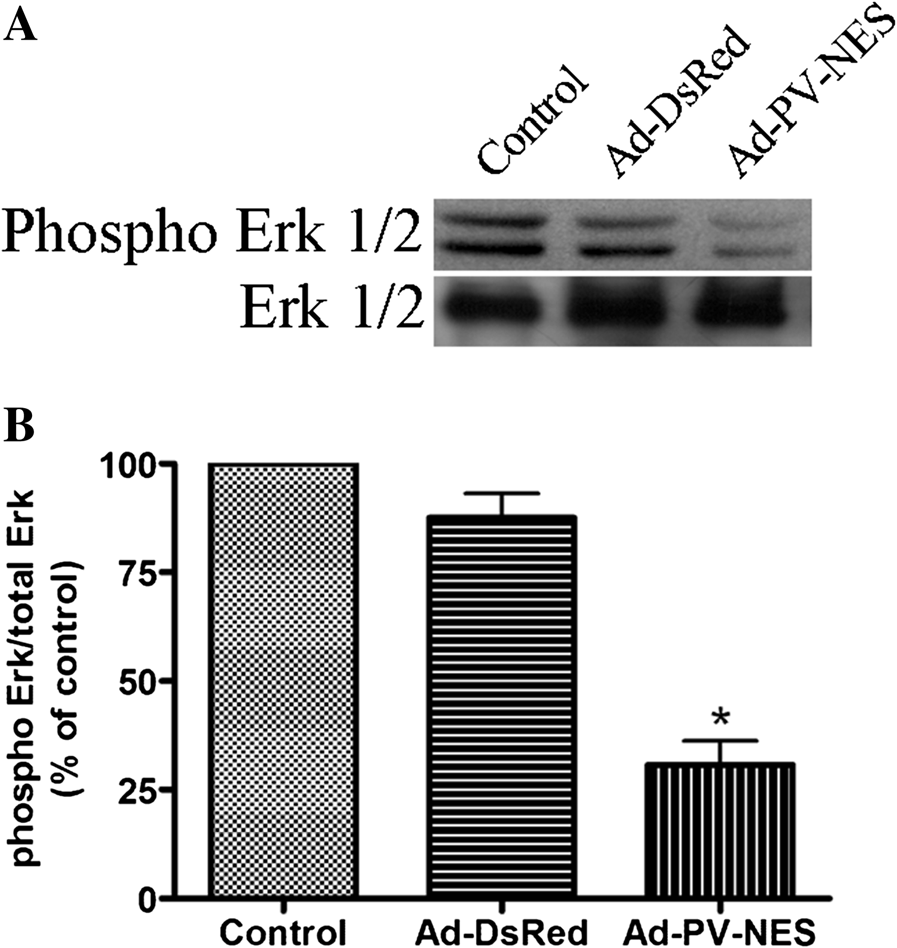
**Cytosolic Ca**^**2+ **^**regulates Erk1/2 phosphorylation. (A)** Immunoblot analysis of cells that were transduced with the Ad-PV-NES-DsRed construct. Erk1/2 phosphorylation was decreased in the presence of the PV-NES-DsRed protein. Total Erk1/2 was used as a loading control. **(B)** Bar-graph summary showing that Ad-PV-NES-DsRed decreased Erk1/2 phosphorylation by 69% ± 5% (*n* = 3). *P < 0.05.

To determine whether the expression of Ad-PV-NES-DsRed specifically affected the G_2_/M transition, we examined the expression of cyclin B1, a G_2_/M-checkpoint protein. The expression of total cyclin B1 was decreased in cells expressing Ad-PV-NES-DsRed (Figure 5A and B). To confirm that Ad-PV-NES-DsRed expression does not inhibit progression through the earlier phases of the cell cycle, we investigated the expression of G_1_/S and S/G_2_ checkpoint proteins, cyclins A/D1/D2/D3, and cyclins E/E2, respectively. Buffering cytosolic Ca^2+^ did not alter the expression of any of these checkpoint proteins (Figure 6A and B). To examine further the effects of cytosolic Ca^2+^ on cell proliferation, the effect of PV-NES on the expression level of the growth-related kinase Erk1/2 was examined, and buffering cytoplasmic Ca^2+^ was found to decrease the level of phospho-Erk1/2 (Figure 6A and B). Together, these observations suggest that Ad-PV-NES-DsRed acts during, but not before, mitosis to regulate MSCs proliferation.

## Discussion

Recent clinical trials that tested MSCs for the treatment of debilitating disorders, such as osteogenesis imperfecta and myocardial infarction, have shown promising results [18]. Numerous preclinical studies have established the therapeutic potential of MSCs in tissue engineering and as cellular protein factories for the delivery of cytokines and anticancer agents [19]. However, although MSC therapy has gained popularity among practitioners and researchers, reports indicated the adverse effects of MSCs, especially in the context of tumor modulation and malignant transformation [6]. Here, we demonstrated that parvalbumin that was targeted to the cytoplasm induced cell-cycle arrest at prophase in rat MSCs. Genetically modified MSCs have been successfully evaluated in animal models for diabetes, skeletal defects, and myocardial infarction [20]. We speculate that genetically modified MSCs, such as the MSCs expressing Ad-PV-NES-DsRed in our study, could be a valuable tool for understanding and controlling MSC proliferation.

The concept that Ca^2+^ signals regulate rat MSC proliferation at the level of the prophase-to-metaphase transition is interesting and novel. We used a cytoplasmic parvalbumin fusion protein as an alternative for intracellular Ca^2+^ chelators, such as BAPTA-AM. PV constructs have the ability to attenuate Ca^2+^ signals specifically to avoid cell death, which is normally observed after long periods of treatment with BAPTA-AM [21]. Increases in the free Ca^2+^ concentration in the cytosol are associated with progression through the cell cycle [22-24]. Increasing evidence suggests that the spatial and temporal patterns of Ca^2+^ signals may determine their specificity for the transitions between each phase of the cell cycle. The cell-cycle arrest observed at prophase in cells expressing Ad-PV-NES-DsRed could be attributed to the ability of this protein to attenuate Ca^2+^ signals specifically.

Previous studies in cell lines and in the liver showed that targeted PV constructs can efficiently alter Ca^2+^ signal profiles and arrest cell-cycle progression at mitosis, but the effects that we observed in the expression of cyclin B1 were not observed in MSCs or other cell models to date [10,17,25]. The cyclin B1/Cdk1 complex regulates many of the dramatic cellular rearrangements observed during mitosis [26]. Cdk1 activation is a multistep process that begins when it binds to its regulatory subunit cyclin B, which increases in expression during G_2_ and peaks in mitosis [27]. Before mitosis, cyclin B/Cdk1 complexes are held in an inactive state by Cdk1 phosphorylation, which is catalyzed by the protein kinases Wee1 and Myt1. Dephosphorylation of Cdk1 is carried out by the protein phosphatase cell-division cycle 25 homolog C (Cdc25C).

Activation of Cdc25C requires the phosphorylation of several sites in the Cdc25C amino-terminal domain [27]. Although the phosphorylation of cyclin B does not appear to regulate the enzymatic activity of the complex, the subcellular localization of the cyclin B1/Cdk1 complex is controlled through the phosphorylation of four serines (Ser 94, 96, 101, and 113 in *Xenopus*) within a region of cyclin B1 that is known as the cytoplasmic retention sequence (CRS) [26]. The phosphorylation of the first two CRS serines (Ser 94 and 96) is catalyzed by Erk in the *Xenopus* system [26]. During mitosis, these cyclin B1 phosphorylations trigger a decrease in export and an increase in import, resulting in nuclear translocation. In late prophase, most cyclin B1/Cdk1 complexes are rapidly translocated from the cytoplasm to the nucleus, after which the nuclear envelope breaks down [27].

In this study, we demonstrated that the attenuation of Ca^2+^ signals by Ad-PV-NES-DsRed decreased cyclin B1 expression and Erk phosphorylation, and we hypothesize that these effects caused cell-cycle arrest at prophase. The immediate-early gene transcription and early phosphorylation of Erk and cAMP response element-binding (CREB) have been reported as crucial events for cell-cycle progression and are dependent on cytosolic Ca^2+^ signaling [10,17,28].

Therefore, this tool may represent a new method to control MSC proliferation *in vitro*. Further research is required to identify the targets of cytosolic Ca^2+^ signals for MSC proliferation.

## Conclusions

Cytoplasmic Ca^2+^ signals are important for the cell-cycle progression of MSCs beyond prophase because of their effects on Erk phosphorylation and cyclin B1 expression.

## Abbreviations

Ad: Adenovirus/adenoviral; DMEM: Dulbecco modified Eagle medium; DsRed: *Discosoma* red fluorescent protein; Erk1/2: Extracellular signal-regulated kinase; FACS: Fluorescence-activated cell sorting; FBS: Fetal bovine serum; MOI: Multiplicity of infection; MSC: Adipose-derived MSC; MSC: Multipotent mesenchymal stromal cell; MTT: 3-(4,5-Dimethylthiazol-2-yl)-2,5-diphenyltetrazolium bromide; NES: Nuclear exclusion signal; PBS: Phosphate-buffered saline; PI: Propidium iodide; PV: Parvalbumin; PV-NES-DsRed: Parvalbumin fusion protein targeted to the cytosol; SEM: Standard error of the mean; TBS: Tris-buffered saline.

## Competing interests

The authors declare that they have no competing interests.

## Authors’ contributions

CSBM performed collection and assembly of data, data analysis, writing of the manuscript, and final approval of the manuscript; JAQAF performed adenovirus amplification and purification, and final approval of the manuscript; NCRC performed qPCR and final approval of the manuscript; CA performed cell isolation and final approval of the manuscript; JLC performed MSC isolation and characterization and final approval of the manuscript; AMG performed study conception and design, provision of study materials, and final approval of the manuscript; MAR performed study conception and design, data analysis and interpretation, provision of study materials, and final approval of the manuscript; DAG performed study conception and design, data analysis and interpretation, provision of study materials, and manuscript writing. All authors read and approved the final manuscript.
